# Pevonedistat (MLN4924): mechanism of cell death induction and therapeutic potential in colorectal cancer

**DOI:** 10.1038/s41420-020-00296-w

**Published:** 2020-07-21

**Authors:** Jennifer Ferris, Margarita Espona-Fiedler, Claudia Hamilton, Caitriona Holohan, Nyree Crawford, Alex J. McIntyre, Jamie Z. Roberts, Mark Wappett, Simon S. McDade, Daniel B. Longley, Victoria Coyle

**Affiliations:** Centre for Cancer Research and Cell Biology, Queen’s University Belfast, Belfast, Northern Ireland BT9 7BL UK

**Keywords:** Colon cancer, Colon cancer

## Abstract

Pevonedistat (MLN4924), a selective inhibitor of the NEDD8-activating enzyme E1 regulatory subunit (NAE1), has demonstrated significant therapeutic potential in several malignancies. Although multiple mechanisms-of-action have been identified, how MLN4924 induces cell death and its potential as a combinatorial agent with standard-of-care (SoC) chemotherapy in colorectal cancer (CRC) remains largely undefined. In an effort to understand MLN4924-induced cell death in CRC, we identified p53 as an important mediator of the apoptotic response to MLN4924. We also identified roles for the extrinsic (TRAIL-R2/caspase-8) and intrinsic (BAX/BAK) apoptotic pathways in mediating the apoptotic effects of MLN4924 in CRC cells, as well as a role for BID, which modulates a cross-talk between these pathways. Depletion of the anti-apoptotic protein FLIP, which we identify as a novel mediator of resistance to MLN4924, enhanced apoptosis in a p53-, TRAIL-R2/DR5-, and caspase-8-dependent manner. Notably, TRAIL-R2 was involved in potentiating the apoptotic response to MLN4924 in the absence of FLIP, in a ligand-independent manner. Moreoever, when paired with SoC chemotherapies, MLN4924 demonstrated synergy with the irinotecan metabolite SN38. The cell death induced by MLN4924/SN38 combination was dependent on activation of mitochondria through BAX/BAK, but in a p53-independent manner, an important observation given the high frequency of *TP53* mutation(s) in advanced CRC. These results uncover mechanisms of cell death induced by MLN4924 and suggest that this second-generation proteostasis-disrupting agent may have its most widespread activity in CRC, in combination with irinotecan-containing treatment regimens.

## Introduction

By regulating protein turnover, the ubiquitin–proteasome system (UPS) plays a crucial role in regulating cellular proliferation and survival, with its inhibition regarded as an attractive anti-cancer strategy^1^. The first-generation proteasome inhibitor, Bortezomib, demonstrated efficacy in treatment of hematological malignancies; however, caused significant Grade 3/4 toxicities and demonstrated a lack of efficacy in solid tumors, as a single agent and in combination with standard-of-care therapies^2^. Second-generation proteasomal inhibitors, including more selective inhibitors, have since been developed with the goal of targeting the UPS in solid cancers. Foremost amongst these agents is Pevonedistat (MLN4924), which specifically targets NEDD8-activating enzyme E1 regulatory subunit (NAE1), which governs the first step of the enzymatic NEDDylation cascade. NEDD8 (neural precursor cell expressed developmentally downregulated protein 8) is a 9 kDa ubiquitin-like protein that is conjugated to substrates in a similar manner to ubiquitin. NEDDylation is required for a number of cellular processes, the best characterized being activation of the Cullin-RING E3 Ligase (CRL) family of E3 ubiquitin ligases^3,4^, which are responsible for ubiquitination of 20% of the proteome.

MLN4924 forms an adduct between the C-terminus of NEDD8 and ATP-binding site of NAE1, resembling the NEDD8-adenylate intermediate formed during the NEDD8–NAE1 interaction^5^. MLN4924 is a potent inhibitor of NEDD8 conjugation, with the rapid deNEDDylation of Cullins-1, -2, -3, -4a, -4b, and -5 observed in vitro following treatment^6^, resulting in CRL inactivation and subsequent accumulation of CRL substrates^7^. Multiple studies have shown that MLN4924 induces cell death, promotes anti-tumor immunity^8^, and inhibits xenograft growth in various pre-clinical models of solid tumors^9^. MLN4924 has also demonstrated activity as monotherapy in clinical trials in hematological malignancies, and studies evaluating the combination of MLN4924 in combination with SoC chemotherapies in hematological malignancies, pediatric cancers, and lung cancer are ongoing^10^.

Colorectal cancer (CRC) has one of the highest Worldwide incidence and mortality rates, with ~1.8 million new cases and ~881,000 deaths occurring per annum^11,12^. In metastatic CRC (mCRC), 5-year overall survival rates are dismal (only ~8% of patients survive beyond 5 years)^13^; thus, new therapeutic approaches are urgently needed. Previous pre-clinical studies in CRC have demonstrated that MLN4924 induces DNA re-replication, cell cycle arrest, and apoptosis in vitro, and impairment of patient-derived xenograft growth in vivo^14^. In this study, we assessed the potential clinical utility of MLN4924 using in vitro models of CRC, focusing on the mechanisms by which it induces cell death as a single agent, and its potential as a combinatorial agent with standard-of-care chemotherapeutics in genetically defined, clinically relevant disease subgroups.

## Results

### Wild-type p53 enhances MLN4924-induced CRC cell death

**Fig. 1:**
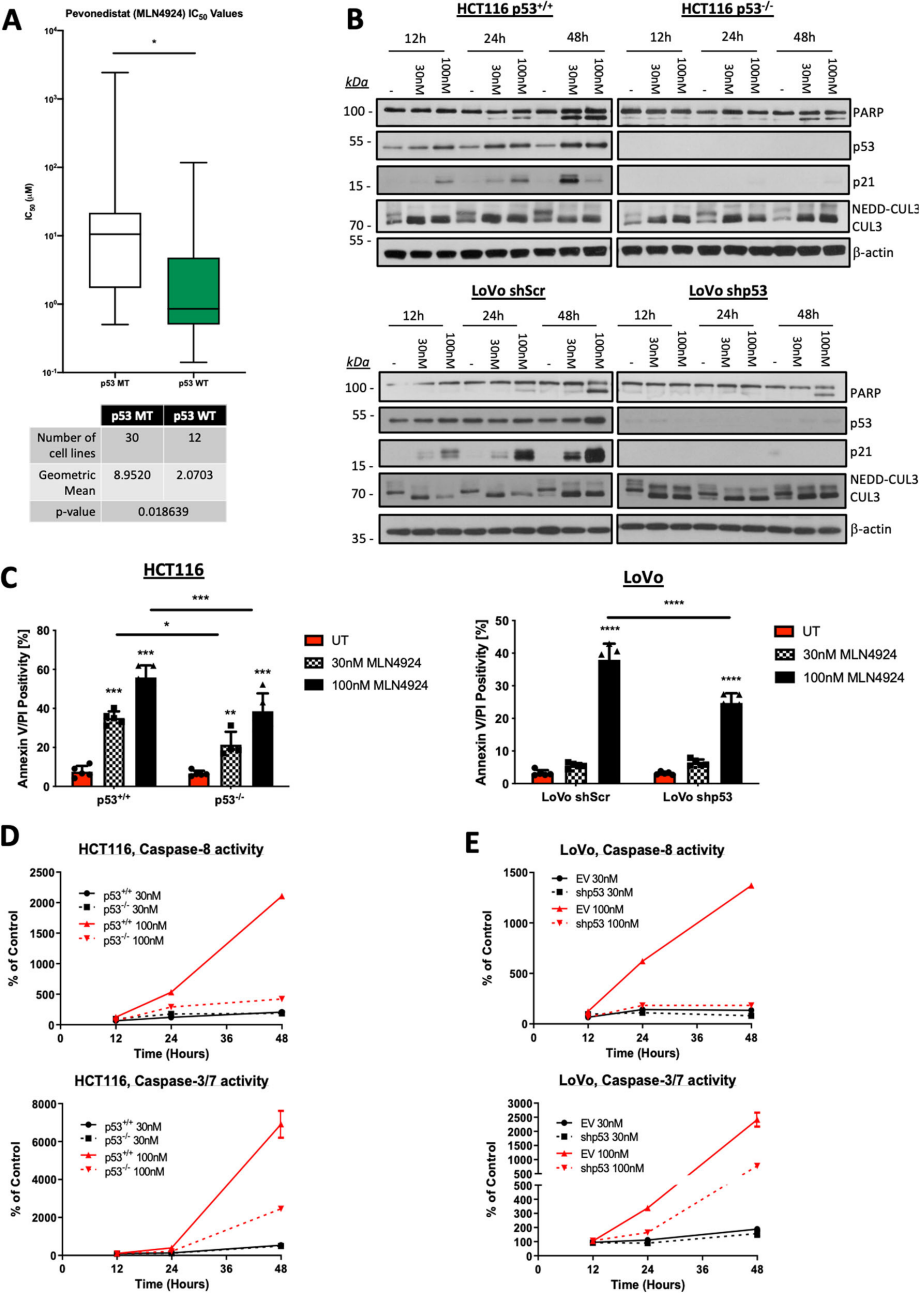
p53 Proficiency enhances CRC cell death induced by MLN4924. **a** Sensitivity of a panel of p53 wild-type and mutant CRC cell lines to MLN4924. Data obtained from the Genomics of Drug Sensitivity in Cancer (GDSC) Project (https://www.cancerrxgene.org/). **b** Western blot analyses of poly-ADP ribose polymerase (PARP), p53, p21, Cullin-3 (CUL3), and β-actin in cells treated with 30 nM and 100 nM MLN4924 for 12, 24, and 48 h. **c** Annexin-V/Propidium Iodide (AV/PI) flow cytometric analysis of HCT116 p53^+/+^ and p53^−/−^, and LoVo shScr and shp53 cell lines treated for 48 h with 30 nM and 100 nM MLN4924. **d**, **e** Caspase-3/7 and -8 activity in HCT116 p53^+/+^ and p53^−/−^, and LoVo shScr and shp53 cell lines treated with 30 nM and 100 nM MLN4924 for 12, 24, and 48 h. An unpaired Student’s T-test (Fig. 1a; GDSC dataset) or Two-way ANOVA multiple comparisons test was used to compute *p*-values for mean ± SD of three independent experiments (Fig. 1c). Asterisks indicate statistically significant changes (*p ≤ 0.05; n.s denotes not significant).

Although it has been demonstrated that MLN4924 has an impact on the apoptotic and DNA damage response machinery in cancer cells, the predominant effects of MLN4924 in CRC described to date relate to its effects on cell cycle progression^15–17^. Although it is evident from a number of studies that NEDDylation inhibition induces cancer cell death, with previous studies indicating a role of CRL substrate NOXA and the initiator caspase-8^18,19^, the manner by which cell death occurs remains largely unresolved. Analysis of pharmacogenomic screening data from Version 2 of the Genomics of Drug Sensitivity in Cancer (GDSC) CRC cell lines indicated that p53 wild-type CRC cell lines (*n* = 12) were significantly more sensitive to MLN4924 than p53 mutant models (*n* = 30; *p* = 0.019; Fig. 1a). Notably, this was not observed in any other cancer types represented in the cell line panel other than cervical cancer (*p* = 0.04; *n* = 7).

Western blot analyses of the effects of MLN4924 on PARP cleavage (a marker of cell death) confirmed that p53-proficient HCT116 and LoVo cells were significantly more sensitive to MLN4924-induced cell death than their respective isogenic p53-deficient daughter cell lines (Fig. 1b). Increased expression of both p53 and its canonical transcriptional target p21 was observed in response to MLN4924, and inhibition of NEDDylation was confirmed by loss of the NEDDylated form of Cullin-3 (Fig. 1b). The contribution of p53 in mediating sensitivity to MLN4924-induced cell death was further confirmed by flow cytometry in both HCT116 and LoVo isogenic models (Fig. 1c). Moreover, MLN4924 significantly increased the activity of caspase-8, and also the executioner caspases of the apoptotic cascade, caspase-3/7, in a p53-dependent manner (Fig. 1d, e). Collectively, these results indicate that although p53-deficient CRC cells can undergo apoptosis in response to MLN4924, the level of cell death induced in p53 wild-type CRC cells is significantly greater.

### Mechanistic insights into MLN4924’s mode-of-action from CCLE and GDSC databases

**Fig. 2:**
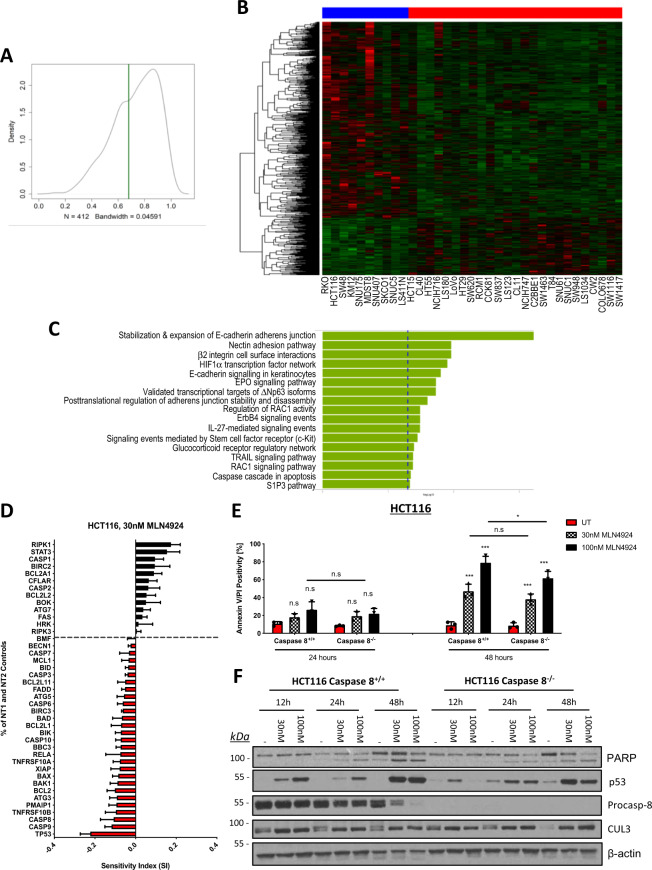
Role of the extrinsic apoptotic pathway in mediating MLN4924-induced cell death. **a** Bimodal distribution of AUC values in solid cell lines from GDSC. **b** Nine hundred and forty-eight differentially expressed genes between 10 CRC cell lines with AUC < 0.68 and 25 cell lines with AUC > 0.68. **c** Pathway analysis of 948 genes from **b**. **d** HCT116 cells were reverse transfected with 10 nM siRNA targeting known regulators of cell death prior to treatment with 30 nM MLN4924. Viability was assessed after 72 h. Sensitivity Index (SI) was used to determine which siRNAs enhance or antagonize the effects of MLN4924, with positive values indicating a sensitizing effect and negative values an antagonistic effect. **e** Annexin-V/PI flow cytometric analyses of HCT116 Caspase-8 CRISPR knockout and matched Empty Vector (EV; Caspase-8^+/+^) cell lines treated for 24 and 48 h with 30 and 100 nM MLN4924. **f** Western blot analysis of PARP, p53, caspase-8, CUL3, and β-actin following 24 and 48 h treatment with 30 and 100 nM MLN4924 in the HCT116 Caspase-8 CRISPR knockout and EV control cell lines. Two-way ANOVA with multiple comparisons test was used to compute *p*-values for mean ± SD of at least three independent experiments. Asterisks indicate statistically significant changes (*p ≤ 0.05, **p ≤ 0.01; n.s denotes not significant).

To further investigate the mechanism-of-action of MLN4924 in CRC, we accessed baseline transcriptomics data from the Cancer Cell Line Encyclopedia (CCLE)^20^ and incorporated it with the cell-viability area under curve (AUC) values from the GDSC^21,22^ data, focusing on solid tumor cell lines. We noted that the density distribution of AUC values had a non-normal distribution (Fig. 2a) and so used the shoulder at AUC = 0.68 to split the data into sensitive and resistant subgroups. The data were then filtered to include only the 35 CRC cell lines (Supplementary Table S1) with both a CCLE and a GDSC profile. Limma^23^ was used to compare the CRC cell lines divided by the 0.68 AUC threshold, identifying 948 genes that were differentially expressed between the two groups (Fold Change [FC] > 2; *p*-value < 0.05; Fig. 2b and Supplementary Table S2). EnrichR Pathway^24^ analysis of this gene set revealed significant signaling pathway differences in MLN4924-sensitive and -resistant models, with enrichment in adherens junction and adhesion in the more sensitive models (Fig. 2c and Supplementary Table S3). There were some compelling standalone markers of resistance (Supplementary Fig. 1A), including multi-drug resistance protein 1 (*ABCB1*) and Ras-related protein Rab-32 (*RAB32*), a marker of endoplasmic reticulum (ER)-, and mitochondrial stress. In terms of cell death signaling, the sensitive models were significantly more primed for caspase activation/apoptosis and TNF-related apoptosis-inducing ligand (TRAIL) signaling pathways; interconnected pathways which are transcriptionally regulated by p53.

### MLN4924 activates extrinsic and intrinsic apoptotic signaling pathway to elicit cell death in CRC

To follow up the transcriptomics-based investigations, we next evaluated the effects of combining an IC_50_ (72 h) dose of MLN4924 (30 nM) with a focused panel of small interfering RNAs (siRNAs) targeting established p53-dependent and -independent regulators of cell death in the HCT116 CRC cell line. Genes were ranked based on Sensitivity Index (SI) values, calculated from measurements of the effects on viability of siRNAs, MLN4924, and their combinations (Fig. 2d). To validate the screen, p53 was included and was indeed identified as the top mediator of sensitivity to MLN4924. In agreement with the pathway analyses, both procaspase-8 (*CASP8*) and TRAIL-R2/DR5 (*TNFRSF10B*) were identified as mediators of response to MLN4924, along with procaspase-9 (*CASP9*) and NOXA (*PMAIP1*). Furthermore, RIPK1 (*RIPK1*), STAT3 (*STAT3*), cIAP1 (*BIRC2*), and FLICE-like inhibitory protein (FLIP; *CFLAR*) were identified as the top-ranked MLN4924 resistance genes (Fig. 2d), whose depletion increased the effects of MLN4924. Notably, three out of four of these proteins (RIPK1, cIAP1, and FLIP) are key components of TRAIL signaling, whereas caspase-8 is the effector caspase for this pathway. Moreover, as demonstrated in Fig. 1d, MLN4924 significantly upregulated the activity of caspase-8, which correlated with an increase in caspase-3 activity (Fig. 1d; Spearman’s *r* = 0.85; *p* = 0.0009). In agreement with this and the siRNA screen results, analysis of HCT116 cells, in which caspase-8 (*CASP8*) expression was knocked-out with CRISPR-Cas9, identified a small inhibitory effect on MLN4924-induced apoptosis, as assessed quantitatively by flow cytometry (Fig. 2e) and qualitatively by PARP cleavage (Fig. 2f).

**Fig. 3:**
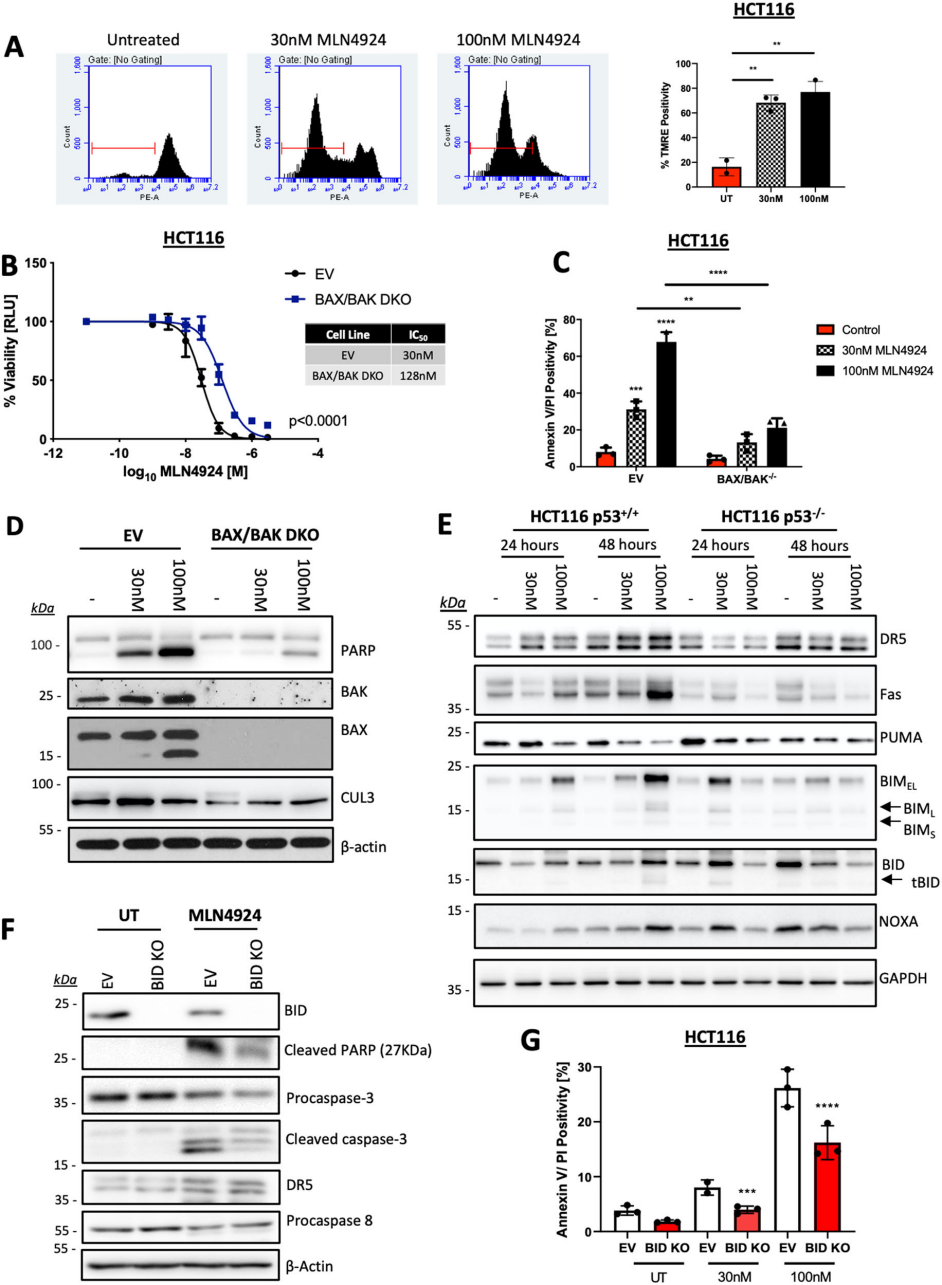
MLN4924 activates the intrinsic apoptotic signaling pathway to elicit cell death. **a** Mitochondrial outer membrane potential (Δψm) was measured following a 48 h treatment with 30 nM or 100 nM MLN4924 via flow cytometry following TMRE staining (25 nM). **b** CellTiter-Glo® cell viability assay in control (EV) and HCT116 BAX/BAK double-knockout (DKO) cells treated for 72 h with MLN4924. Values were normalized to untreated control. IC_50_ values were calculated via a nonlinear regression model, based on the mean ± SD of three independent experiments. **c** Annexin-V/PI flow cytometry analysis of HCT116 EV and BAX/BAK DKO cells treated with 30 and 100 nM MLN4924 for 48 h. **d** Western blot analyses of PARP, BAK, BAX, CUL3, and β-actin expression in HCT116 EV and BAX/BAK DKO cells treated with 30 and 100 nM MLN4924 for 48 h. **e** Western blot analyses of DR5, Fas, BID, BIM, NOXA, PUMA, and β-actin expression following 24 and 48 h exposure of HCT116 p53^+/+^ and p53^−/−^ cells to 30 and 100 nM MLN4924. **f** Western blot analyses of BID, PARP, caspase-3, DR5, procaspase-8, and β-actin following treatment of HCT116 EV BID CRISPR KO cells treated with 100 nM MLN4924 for 48 h. **g** High-content annexin-V/PI analysis of HCT116 EV and BID knockout (KO) cells following treatment with MLN4924 for 48 h. An unpaired Student’s T-test (Fig. 3a) or Two-way ANOVA with multiple comparisons test was used to compute *p*-values for mean ± SD of three independent experiments. Asterisks indicate statistically significant changes (*p ≤ 0.05, **p ≤ 0.01, and ***p ≤ 0.001; n.s denotes not significant).

Procaspase-9, the initiator of the intrinsic mitochondrial apoptotic pathway, was also identified in our siRNA screen, along with the two main effectors of mitochondrial outer membrane permeabilization (MOMP), BAX and BAK, which lie upstream of procaspase-9 (Fig. 2d). The role of intrinsic (mitochondrial) apoptosis in mediating a cell death response to MLN4924 was confirmed by loss of mitochondrial membrane potential (ΔΨm) following 48 h MLN4924 treatment (Fig. 3a). The role of intrinsic apoptosis signaling in MLN4924-induced cell death was further assessed using a BAX/BAK double knockout (DKO) HCT116 daughter cell line, which was significantly more resistant to MLN4924-induced effects on cell viability (<0.001; Fig. 3b) and cell death, as assessed by flow cytometry (Fig. 3c) and PARP cleavage (Fig. 3d). Moreover, in the matched EV HCT116 cells, both BAX and BAK were upregulated in response to MLN4924 (Fig. 3d). Activation of MOMP by BAX/BAK is triggered by the BH3-only protein subset of the Bcl-2 family, which either directly or indirectly promote BAX/BAK oligomerization in the outer mitochondrial membrane, leading to pore formation^25^. The BH3-only proteins BIM and NOXA were both significantly upregulated in response to MLN4924 in a predominantly p53-dependent manner, although PUMA was downregulated. BID was also upregulated at 48 h in response to MLN4924 and a band corresponding to the MOMP-promoting form of BID (tBID) was also visible at this time point (Fig. 3e). We also found that MLN4924 significantly enhanced the expression of TRAIL-R2/DR5 in CRC cells, as well as Fas/CD95, both canonical p53 target genes (Fig. 3e and Supplementary Fig. 3A–C). Depleting either BID or NOXA with siRNA partially inhibited MLN4924-induced apoptosis; however, depleting either PUMA or BIM had no effect despite the latter’s potent upregulation (Supplementary Fig. 3F, G). NOXA (*PMAIP1*) was also identified in the siRNA screen (Fig. 2d) and, although the effects of BID silencing were marginal in the siRNA screen, its importance is consistent with caspase-8 activation identified in Fig. 2, as canonically, BID is activated by caspase-8^26^. In support of this analysis, a BID CRISPR-Cas9-knockout model indicated that MLN4924-induced apoptosis was significantly abrogated in the absence of BID (Fig. 3f, g).

### FLIP downregulation enhances sensitivity to MLN4924 in a p53-, caspase-8-, and TRAIL-R2-dependent manner

**Fig. 4:**
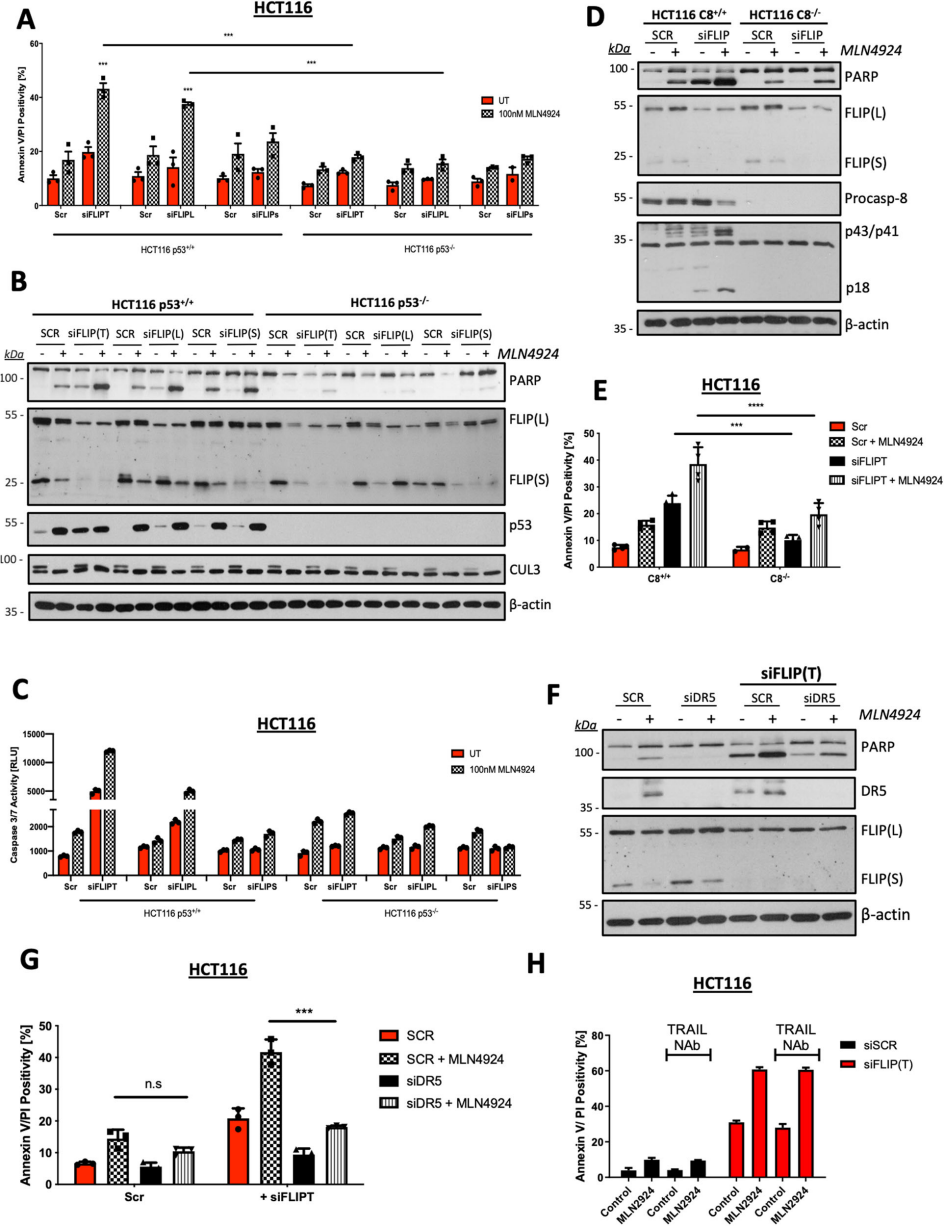
FLIP downregulation enhances sensitivity to MLN4924 in a p53-, caspase-8-, and TRAIL-R2-dependent manner. **a** Annexin-V/PI flow cytometry analysis of HCT116 p53^+/+^ and p53^−/−^ cell lines pre-treated with 10 nM scrambled control (SCR) siRNA, FLIP siRNA targeting both FLIP(L) and FLIP(S) (siFLIP(T)), FLIP(L)-specific siRNA (siFLIP(L)), and FLIP(S)-specific siRNA (siFLIP(S)) for 6 h, followed by 24 h treatment with 100 nM MLN4924. **b** Western blot analyses of PARP, FLIP, p53, CUL3, and β-actin following transfection of HCT116 p53^+/+^ and p53^−/−^ cell lines with 10 nM SCR, siFLIP(T), siFLIP(L), or siFLIP(S) siRNAs and 24 h treatment with 100 nM MLN4924. **c** Caspase-3/7 activity in HCT116 cells co-treated with 10 nM SCR, siFLIP(T), siFLIP(L), or siFLIP(S) siRNAs 100 nM MLN4924 for 24 h. **d** Western blot analyses of PARP, FLIP, Caspase-8, CUL3, and β-actin following transfection of HCT116 CASP8 (C8)^+/+^ and CASP8 (C8)^−/−^ cell lines with 10 nM SCR or siFLIP(T) siRNAs and 24 h treatment with 100 nM MLN4924. **e** Flow cytometry analysis of apoptosis in CASP8 (C8)^+/+^ and CASP8 (C8)^−/−^ cell lines transfected with 10 nM SCR or siFLIP(T) siRNAs and treated with 100 nM MLN4924 for 24 h. **f** Western blot analyses of PARP, DR5 (TRAIL-R2), FLIP, and β-actin expression in HCT116 parental cell lines. Cells were pre-treated with DR5-specific siRNA for 24 h, reseeded, and transfected with 10 nM SCR or siFLIP(T) prior to 24 h treatment with 100 nM MLN4924. **g** Annexin-V/PI flow cytometry analysis of HCT116 cells pre-treated with control (SCR) or DR5-specific siRNA (siDR5) for 24 h, reseeded, and transfected with SCR or siFLIP(T) siRNAs prior to 24 h treatment with 100 nM MLN4924. **h** High-content annexin-V/PI analysis of HCT116 cells transfected with 10 nM FLIP(T)-specific siRNA and treated with 100 ng/mL of TRAIL-neutralizing antibody ± 100 nM MLN4924 for 24 h. Two-way ANOVA with multiple comparisons test was used to compute p-values for mean ± SD of three independent experiments. Asterisks indicate statistically significant changes (*p ≤ 0.05, **p ≤ 0.01, and ***p ≤ 0.001; n.s denotes not significant).

In the apoptosis-focused siRNA screen, downregulation of the Fas-Associated Via Death Domain (FADD)-binding partner and procaspase-8 paralog and regulator FLIP enhanced the effects of MLN4924, as did the known upstream regulators of FLIP expression RIPK1 and cIAP1 (via nuclear factor-κB)^27,28^, and STAT3^29^ (Fig. 2d). Consistent with this, siRNAs targeting both FLIP splice forms, FLIP-Long (FLIP(L)) and FLIP-Short (FLIP(S)), significantly enhanced MLN4924-induced apoptosis, but only in the HCT116 p53 wild-type model (Fig. 4a), an effect also observed in the LoVo paired cell lines (Supplementary Fig. 2A). Protein levels of FLIP(S) were downregulated in response to MLN4924 in both p53-proficient and -deficient models; FLIP(L) downregulation was also observed, but only in the p53-deficient models (Fig. 4b and Supplementary Fig. 2B). The downregulation of FLIP(S) in response to MLN4924 may explain why specific downregulation of FLIP(L) was sufficient to enhance MLN4924-induced apoptosis in the p53 wild-type models, particularly in the HCT116 model, where MLN4924-induced FLIP(S) downregulation was more profound (Fig. 4a, b and Supplementary Fig. 2A, B). Caspase-3/7 activity was in line with the cell death induced in response to co-treatment with siFLIP and MLN4924 (Fig. 4c and Supplementary Fig. 2C). Notably, enhancement of apoptosis induced by FLIP depletion in combination with MLN4924 was clearly caspase-8-dependent, as apoptosis induction and PARP cleavage were abrogated in the caspase-8-deficient daughter cell line (Fig. 4d, e).

The upstream regulator of caspase-8-mediated apoptosis, TRAIL-R2 (DR5, *TNFRSF10B*), was also identified in the pathway analysis (Fig. 2c) and siRNA screen as a potential mediator of response to MLN4924 (Fig. 2d). Moreover, MLN4924 treatment led to upregulation of DR5 expression (Fig. 3e and Supplementary Fig. 3A). In HCT116 cells, siRNA-mediated TRAIL-R2 downregulation abrogated PARP cleavage induced by MLN4924 alone (Fig. 4f), indicating a role for the receptor in mediating MLN4924-induced apoptosis; no PARP cleavage was observed in response to MLN4924 alone in the LoVo model (which is also more resistant in the GDSC panel) at this concentration and time point (Supplementary Fig. 3C). However, in both cell lines, the enhanced cell death induced by MLN4924 in FLIP-depleted cells was also TRAIL-R2-dependent (Fig. 4f, g and Supplementary Fig. 3C, D). Notably, however, these effects were not dependent on TRAIL-R2 ligation, as the cell death induced by MLN4924 in FLIP-depleted cells was not rescued by co-treatment with a TRAIL-neutralizing antibody (Fig. 4h and Supplementary Fig. 3E). Collectively, these results indicate that when FLIP-mediated inhibition is overcome, MLN4924 activates p53-dependent, TRAIL-R2-, and caspase-8-mediated apoptosis in a ligand-independent manner.

### MLN4924 exhibits p53-independent synergy with SN38

**Fig. 5:**
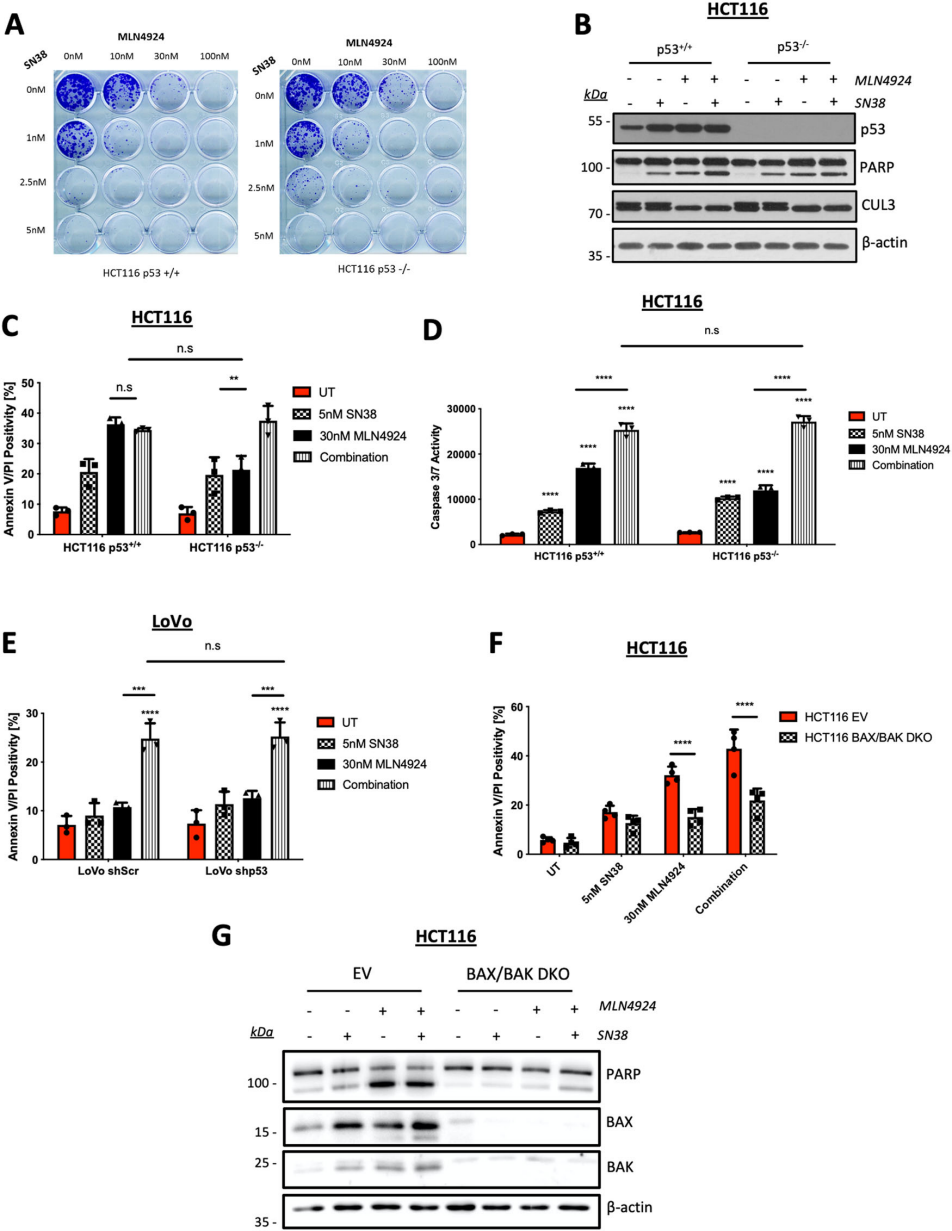
MLN4924 exhibits p53-independent synergy with SN38. **a** Clonogenic assay in HCT116 p53^+/+^ and p53^−/−^ cells co-treated with MLN4924 and SN38 for 24 h prior to media replenishment. Colonies were allowed to form for 7 days prior to fixing and staining with Crystal Violet. **b** Western blot analysis of p53, PARP, CUL3, and β-actin following 48 h treatment of HCT116 p53^+/+^ and p53^−/−^ cells with 5 nM SN38 and 30 nM MLN4924, single agent, and in combination. **c** Annexin-V/PI flow cytometric analysis of HCT116 p53^+/+^ and p53^−/−^ cell lines treated for 48 h with 5 nM SN38 and 30 nM MLN4924 alone or in combination. **d** Caspase-3/7 activity in HCT116 p53^+/+^ and p53^−/−^ cells treated with 5 nM SN38, 30 nM MLN4924, or a combination of both compounds for 48 h. **e** Annexin-V/PI flow cytometric analysis of LoVo shScr and shp53 cell lines treated for 48 h with 5 nM SN38 and 30 nM MLN4924 alone or in combination. **f** Annexin-V/PI flow cytometric analysis of HCT116 BAX/BAK CRISPR-knockout and matched Empty Vector (EV) cell lines treated for 48 h with 5 nM SN38 and 30 nM MLN4924 alone or in combination. **g** Western blot analysis of PARP, BAX, BAK, and β-actin in the HCT116 BAX/BAK CRISPR-knockout and matched EV cell lines treated for 48 h with 5 nM SN38 and 30 nM MLN4924 alone or in combination. Two-way ANOVA with multiple comparisons test was used to compute *p*-values for mean ± SD of three independent experiments. Asterisks indicate statistically significant changes (*p ≤ 0.05, **p ≤ 0.01, and ***p ≤ 0.001; n.s denotes not significant).

The above data indicate that MLN4924 can prime the mitochondria for apoptosis by upregulating the levels of NOXA, BAX, BAK, and BID. Moreover, the importance of wild-type p53 in mediating the apoptotic effects of MLN4924 is consistent with its role as a transcriptional activator of the genes encoding NOXA, BAX, and TRAIL-R2^30–32^. As inefficient apoptosis induction is a major cause of chemotherapy resistance^33^, we hypothesized that MLN4924 may thereby enhance the effectiveness of standard-of-care CRC chemotherapeutic agents in a p53-dependent manner. Thus, we performed a series of clonogenic survival assays to determine the efficacy of MLN4924 in combination with the backbone chemotherapeutic agent for CRC, 5-fluorouracil (5FU), and in combination with the two other main chemotherapeutics used to treat this disease, oxaliplatin and irinotecan. Given the role of p53 in mediating the effects of single-agent MLN4924, we assessed activity in p53 wild-type and p53-deficient HCT116 models. Surprisingly, there was no significant synergy between MLN4924 and either 5FU or oxaliplatin regardless of the p53 status in HCT116 cells (Supplementary Fig. 4A–F). However, there was a clear interaction between MLN4924 and the active metabolite of irinotecan, SN38, at the lowest concentrations used (Fig. 5a); notably, this effect was clearly p53-independent. These results were confirmed by Western blot analyses, AV/PI flow cytometry, and caspase activity assays (Fig. 5b–d), and similar results were obtained in the LoVo model (Fig. 5e), although significant p53-independent enhancement of 5FU-induced apoptosis and p53-dependent enhancement of oxaliplatin-induced apoptosis in MLN4924 co-treated cells were also observed in this cell line (Supplementary Fig. 5A, B).

The interaction between MLN4924 and SN38 was all the more notable as, similar to MLN4924, the effects of SN38 in a wider panel of CRC models was also p53-dependent, with p53 wild-type models significantly more sensitive (Supplementary Fig. 5C). To investigate the mechanism of action behind this synergy, we assessed the impact of the combination therapy in BAX/BAK- and BID-deficient models. A lack of BAX/BAK almost completely attenuated apoptosis induction (Fig. 5f, g), indicating that the combination drives cell death primarily via the intrinsic apoptosis pathway.

## Discussion

Essential for cellular homeostasis, controlled protein turnover is critical for most, if not all, biological processes, including cell cycle progression, cell death, gene expression, and signal transduction. The UPS regulates turnover of the majority of intracellular proteins via interplay between E1 ubiquitin-activating enzymes, E2 ubiquitin-conjugating enzymes, and E3 ubiquitin ligases, which catalyze the transfer of the posttranslational modifier, ubiquitin, onto substrate proteins. The functionality of the largest family of E3 ligases, the CRLs, is controlled by the ubiquitin-like modifier, NEDD8, with NEDDylation of Cullin proteins required to activate the CRL complex. Similar to the ubiquitin-conjugation cascade, NEDD8 conjugation involves the interplay of three enzymes (E1, E2, and E3).

Recognition that the process of malignant transformation profoundly affects protein homeostasis has led to the development of therapeutics that exploit tumor-selective vulnerability to the disruption of proteostasis. Although approved for the treatment of multiple myeloma, the proteasomal inhibitor Bortezomib has shown little anti-tumor efficacy in solid tumors and causes substantial toxicities. The development of more selective, targeted inhibitors of protein degradation aims to reduce the side effects associated with global proteasomal inhibition. MLN4924 is currently undergoing Phase I and Phase II clinical trials in hematological malignancies and some solid tumors (e.g., non-small cell lung carcinoma, melanoma, liver cancer, and mesothelioma), both as a single agent and in combination with chemotherapies, including platinums and taxanes^10^.

A growing number of studies have demonstrated MLN4924’s potential value in the treatment of CRC. For example, Zhang et al.^34^ observed that MLN4924 was a potent radio-sensitizing agent in vitro, enhancing G2/M arrest and apoptosis in HT29 and HCT116 CRC cell lines. This effect was rescued by p27 knockdown, suggesting that this well-established CRL cell cycle regulatory protein is involved in mediating the impact of MLN4924 in response to radiation in these models. A single-agent study in 2009 by Soucy et al.^9^ discovered that MLN4924 deregulated S-phase DNA synthesis, promoted accumulation of DNA damage, and subsequently activated apoptosis in HCT116 cells. The inhibitor was also established to be well tolerated in vivo and caused potent xenograft growth inhibition^9^. Further study demonstrated that the disruption of DNA synthesis induced by MLN4924 treatment was due to stabilization of the CRL substrate Cdt1, a DNA replication factor that induces DNA re-replication upon MLN4924 exposure^35^. Moreover, Picco et al.^14^ investigated response to MLN4924 in 122 CRC cell lines and identified a transcriptional signature of sensitivity. This signature was able to predict response to treatment in patient-derived xenograft models; however, the response observed was a stabilization of tumor growth rather than a reduction^14^, suggesting that MLN4924 may be most effective in combination with other agents as a treatment strategy for CRC.

MLN4924 has been shown to stabilize p53 through its capacity to induce ribosomal stress; e.g., inhibition of RPL11 NEDDylation enables its translocation from ribosomes to impede the MDM2–p53 interaction, leading to p53 stabilization^36^. Indeed, we observed p53 stabilization in response to MLN4924 and found that p53-deficient CRC models were more resistant to MLN4924-induced apoptosis, although significant cell death was still observed in p53-deficient models. This was supported by data from the GDSC online database, which indicated that p53 wild-type CRC models were overall significantly more sensitive to MLN4924 than p53 mutant models. Notably, this was also the case in only one other tumor type (cervical cancer), suggesting that p53’s role in mediating the cytotoxic effects of MLN4924 is tumor type-dependent.

NOXA has previously been reported to mediate MLN4924-induced cell death^18,37^ and our results (Supplementary Fig. 3F, G) would support this. However, we also found that another BH3-only protein BID plays an important role in mediating MLN4924-induced apoptosis in CRC cells. BID is activated downstream of caspase-8 in the extrinsic apoptotic pathway; consistent with this, we identified a role for caspase-8 in mediating cell death in response to MLN4924 alone. This is in agreement with a study in esophageal cancer, which demonstrated the interplay between ER stress and apoptosis in response to MLN4924. This study found that ATF4 was stabilized upon MLN4924 treatment, which subsequently activated the ER stress mediator CCAAT-enhancer-binding protein homologous protein (CHOP), leading to the upregulation of the death receptor TRAIL-R2^19^ and activation of caspase-8 and BID. Consistent with the roles of NOXA and BID, we found that co-deletion of BAX and BAK, both of which were upregulated in response to MLN4924, protected CRC cells from MLN4924-induced cell death, indicating an important role for intrinsic apoptosis in this response. With regard to p53-mediated sensitivity, the transcription factor is also capable of transcriptionally activating BAX, as well as directly activating BAX and BAK at the mitochondrial outer membrane^31,38,39^ to initiate MOMP.

FLIP is a well-established apoptosis-regulatory protein implicated in resistance to standard-of-care therapies^40–42^, regulating procaspase-8 processing at the DISC and related complexes in a splice form-specific manner^43^. FLIP silencing in combination with short-term MLN4924 treatment significantly enhanced cell death in a manner that was highly dependent on p53, TRAIL-R2, and caspase-8. Moreover, MLN4924 upregulated TRAIL-R2 expression in both p53 wild-type models (HCT116 and LoVo). These results indicate that FLIP silencing induces p53-dependent, TRAIL-R2-, and caspase-8-mediated apoptosis upon treatment with MLN4924. However, ligation of TRAIL-R2 by its canonical ligand TRAIL was not required. TRAIL-R2-dependent and TRAIL-independent cell deaths in response to chemotherapy^44^ and during ER stress^45^ have been described, with the latter shown to be triggered by binding of misfolded proteins to TRAIL-R2 at the ER^46^, a result consistent with the reported impact of MLN4924 on ER stress (and consistent with our CCLE/GDSC analyses). At the protein level, MLN4924 treatment downregulated FLIP(S) in both p53-proficient and -deficient models, but only downregulated FLIP(L) in p53-deficient models (Fig. 4b and Supplementary Fig. 2B). MLN4924 has previously been implicated in promoting JNK-mediated degradation of FLIP(L) and FLIP(S)^47^. Our results suggest that the residual FLIP(L) expression in MLN4924-treated cells is a major mechanism of resistance to this agent in p53 wild-type CRC cells.

In the metastatic disease setting (mCRC), CRC patients are currently treated with 5FU-based regimens (plus oxaliplatin, FOLFOX; or plus irinotecan, FOLFIRI); however, 5-year overall survival rates are only ~8%^13^. In absence of a predictive biomarker, novel therapeutics have had modest effects as monotherapy for the treatment of mCRC. Hence, there is a critical need to develop new therapeutic approaches for mCRC. The study reported by Picco et al.^14^ did not find a correlation between response to MLN4924 monotherapy and clinically relevant CRC molecular drivers (*KRAS* mutation, *BRAF* mutation, and microsatellite instability); however, transcriptional profiling of a cohort of mCRC tumors suggested that high-grade mucinous carcinomas could be a responsive subgroup^14^. Our data suggest that both p53 wild-type and particularly mutant mCRC may be responsive to MLN4924 in combination with irinotecan-containing regimens (FOLFIRI), whereas combinations with oxaliplatin (FOLFOX) would be most effective in p53 wild-type mCRC. Thus, our data support further evaluation of the second-generation proteostasis-disrupting agent MLN4924 in CRC; in particular, combinations of MLN4924 with irinotecan-containing chemotherapy regimens may be particularly effective in chemo-refractory p53 mutant CRC.

## Materials and methods

### Compounds

MLN4924 [I-502] (Pevonedistat) was obtained from Boston Biochem (Cambridge, MA). SN38, oxaliplatin, and 5FU were obtained from Belfast City Hospital, Belfast Health and Social Care Trust, Belfast. Human TRAIL-neutralizing antibody was purchased from R&D Systems (Minneapolis, MN).

### Cell lines and cell culture

HCT116 p53^+/+^ and p53^−/−^ cell lines were obtained from the Vogelstein Laboratory (Johns Hopkins University School of Medicine, Baltimore). LoVo shScr and shp53 were generated by transducing the parental model with retroviral pSUPER vectors expressing control or p53 short hairpin RNA under puromycin selection (0.5 µg/mL). HCT116 BAX/BAK DKO cells were obtained from Professor Markus Rehm (University of Stuttgart, Germany). HCT116 caspase-8 CRISPR cells were obtained from Professor Galit Lahav (Department of Systems Biology, Harvard Medical School, Boston, MA)^48^. All HCT116-derived cell lines were cultured in McCoy’s 5A Modified Medium (ATCC, LGC Standards, Middlesex, UK) supplemented with 10% fetal bovine serum (Invitrogen, Carlsbad, CA). LoVo cells were cultured in Dulbecco’s modified Eagle’s medium (ATCC, LGC Standards, Middlesex, UK) with 10% fetal bovine serum, at 37 °C in a humidified atmosphere of 5% CO_2_. Cell lines in culture were tested at least monthly for Mycoplasma using the Lonza MycoAlert™ kit.

### Western blot analysis

Whole-cell protein lysates were prepared and Western blotting was carried out as previously described^49^. PARP, FAS, DR5, BIM, BID, NOXA, PUMA, and procaspase-3-specific antibodies were obtained from Cell Signaling Technology (Danvers, MA). Cullin-3-specific antibody was obtained from BD Biosciences (Santa Jose, CA); p53- and p21-specific antibodies were obtained from Santa Cruz Technologies (Dallas, TX); FLIP-specific antibody (NF6) was obtained from Adipogen (San Diego, CA). Caspase-8 antibody was from Enzo Life Sciences (Farmingdale, NY). Secondary horseradish peroxidase-conjugated antibodies from Cell Signaling Technology (Danvers, MA) were used for detection on a G-Box digital developer (Syngene Cambridge, UK). Antibody catalog numbers are listed in Supplementary Table S4.

### Flow cytometry

Detection of cell-surface DR5 and Fas expression was conducted using the BD Accuri C6 flow cytometer, with analyses completed on the Accuri C6 PLUS software (BD Biosciences, San Diego, CA), and cells stained using Phycoerythrin-conjugated anti-DR5 or anti-FAS antibody compared with an isotype control antibody (IgG) (Biolegend, San Diego, CA). Annexin-V/Propidium Iodide flow cytometry was carried out on a BD LSRII flow cytometer (BD Biosciences, San Diego, CA) using fluorescein isothiocyanate (FITC)-tagged Annexin-V (BD Biosciences) and Propidium Iodide (Sigma-Aldrich, MO). Loss of mitochondrial outer membrane potential was quantified following staining with 25 nM Tetramethylrhodamine ethyl ester (Sigma-Aldrich, MO) for 15 min prior to flow cytometric analyses on the BD Accuri C6 flow cytometer.

### High-content microscopy

Cells were seeded into a 96‐well glass‐bottomed plate (Cellvis) and left to adhere overnight. After treatments, cells were incubated with 10× Annexin-V Binding Buffer, 1:1000 FITC Annexin-V (BD Pharmingen, San Diego, CA), 0.33 μg/mL Propidium Iodide (Sigma-Aldrich, MO), and 1.33 μg/mL Hoechst 33342 (Thermo Fisher Scientific, Waltham, MA) for 20 min at room temperature. The plate was analyzed on the ArrayScanTM XTI HCA Reader, integrated with the CrESTTM X‐LightTM Confocal Scan Head (Thermo Fisher Scientific, Waltham, MA).

### siRNA transfections

All siRNAs, apart from those targeting FLIP (Eurofins Scientific, Luxembourg), were obtained from Dharmacon (Chicago, IL) and transfections carried out using Lipofectamine RNAiMAX (Life Technologies, Carlsbad, CA), as previously described^50^.

### Cell death siRNA library screen

A Dharmacon siGENOME SMARTpool siRNA library was generated to target 41 cell death regulators. siRNA duplexes were seeded onto 384-well source plates, which were stored at −20 °C until required. Using an automatic acoustic liquid handler, the Labcyte ECHO (Labcyte, San Jose, CA), siRNAs were transferred from the source plate to a 384-well destination plate. Lipofectamine RNAiMAX Transfection reagent was mixed in a 1:167 ratio with OptiMEM reduced-serum media (Thermo Fisher Scientific, Waltham, MA) and 10 µL was added to each well and incubated for 20 min to allow for liposome formation. Nine hundred cells were seeded into each well, each containing a final concentration of 10 nM siRNA, and plates were incubated overnight to allow cells to adhere and transfection to occur. The following day, the transfection mix was removed and 50 µL relevant antibiotic-free media was added containing appropriate MLN4924 concentrations and incubated for the required time. Transfection conditions, cell seeding densities, drug concentrations, and time points were optimized in pilot experiments with specific transfection controls (untransfected, non-targeting siRNA, and PLK1 siRNA) used to confirm adequate transfection efficiency.

### Caspase and cell-viability assays

Caspase activity was measured using Caspase-Glo® assay reagents and cell viability assessed by Cell Titer-Glo® (Promega, Madison, WI).

### Clonogenic survival assay

Cells were seeded at a density of 500 cells per well and treated for 24 h before media replacement. Following 7–10 days, colony formation was assessed via Crystal Violet staining and quantified by resuspension of the dye absorbed by the colonies using 1 M sodium citrate:50% ethanol solution, with absorbance measured at 570 nm on the Biotrak II microplate reader.

### Statistical analysis

Statistical significance was calculated from distinct technical replicates (*n* ≥ 3), either by Student’s *T*-test (two-tailed, two-sample equal variance on unpaired data) or two-way analysis of variance in GraphPad Prism 8. Graphs were plotted as means with error bars represented as SEM; statistical significance was denoted as follows: ****p < 0.0001, ***p < 0.001, **p < 0.01, *p < 0.05, ns = *p* > 0.05. Experimental phenotypes were confirmed in at least three independent experiments.

SI was a statistical method utilized to interpret raw data obtained from the cell death siRNA screen. SI estimates the combined influence of siRNA knockdown on drug sensitivity and the individual drug and siRNA effects.

SI = (Rc/Cc*Cd/Cc) − (Rd/Cc), where

Rc: average viability in untreated wells transfected with siRNA

Rd: average viability in drug-treated wells with siRNA

Cc: average viability in untreated wells with control siRNA

Cd: average viability in drug-treated wells with control siRNA

The SI value ranges from −1 to 1, with positive values indicating a sensitizing effect and negative values indicating an antagonizing effect.

## Supplementary information

Supplementary Figure 1

Supplementary Figure 2

Supplementary Figure 3

Supplementary Figure 4

Supplementary Figure 5

Supplementary Table S1

Supplementary Table S2

Supplementary Table S3

Supplementary Table S4

## Data Availability

The data supporting the findings of the study are available from the corresponding author on reasonable request.
