# Supplementary figures and images for: Pevonedistat (MLN4924): mechanism of cell death induction and therapeutic potential in colorectal cancer
Source: Cell Death Discov. 2020 Jul 21;6:61. doi: 10.1038/s41420-020-00296-w (PMC7374701; doi:10.1038/s41420-020-00296-w)

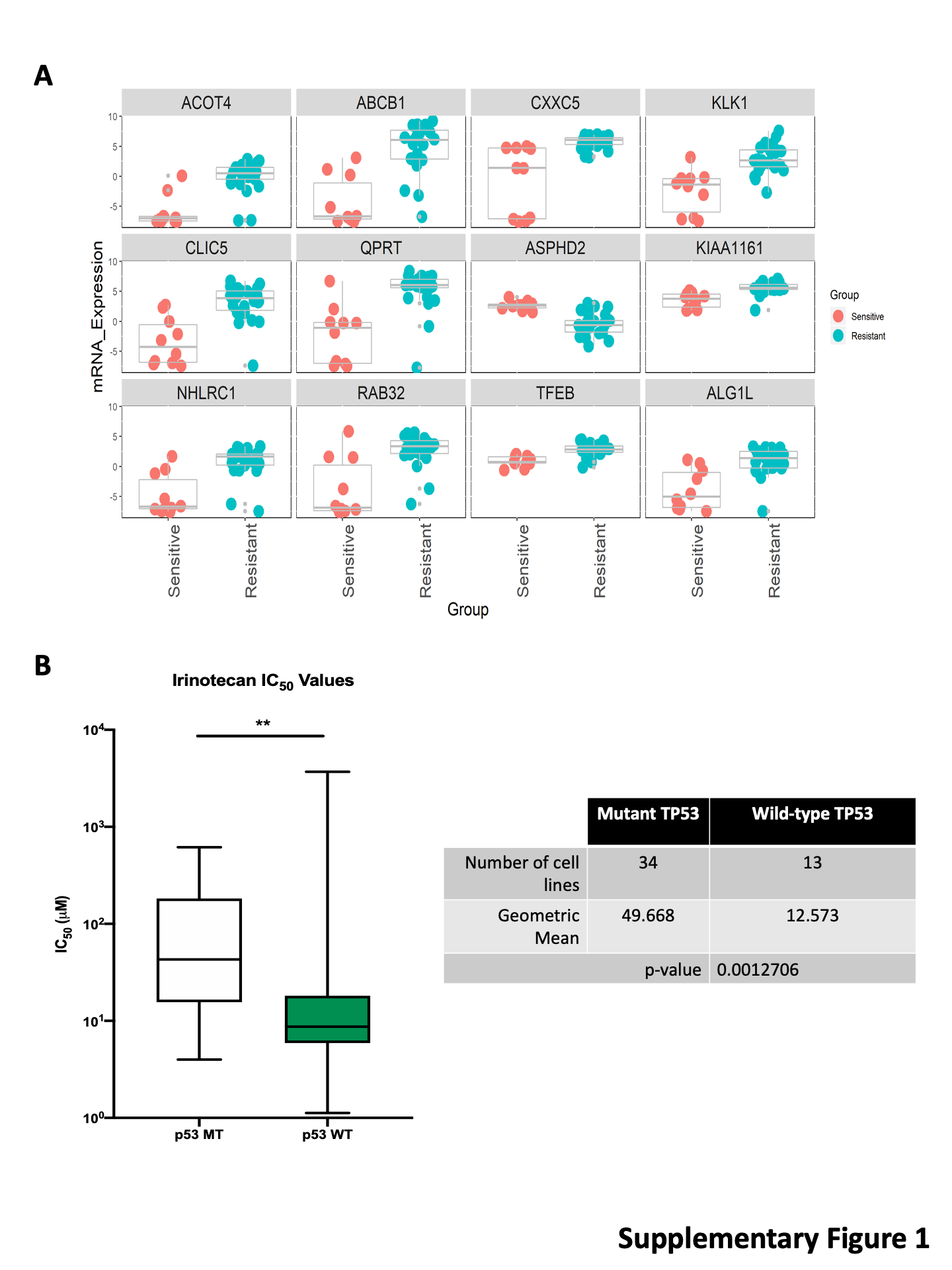

Supplement: Supplementary file 1 — Supplementary Figure 1 [file 41420_2020_296_MOESM1_ESM.png]

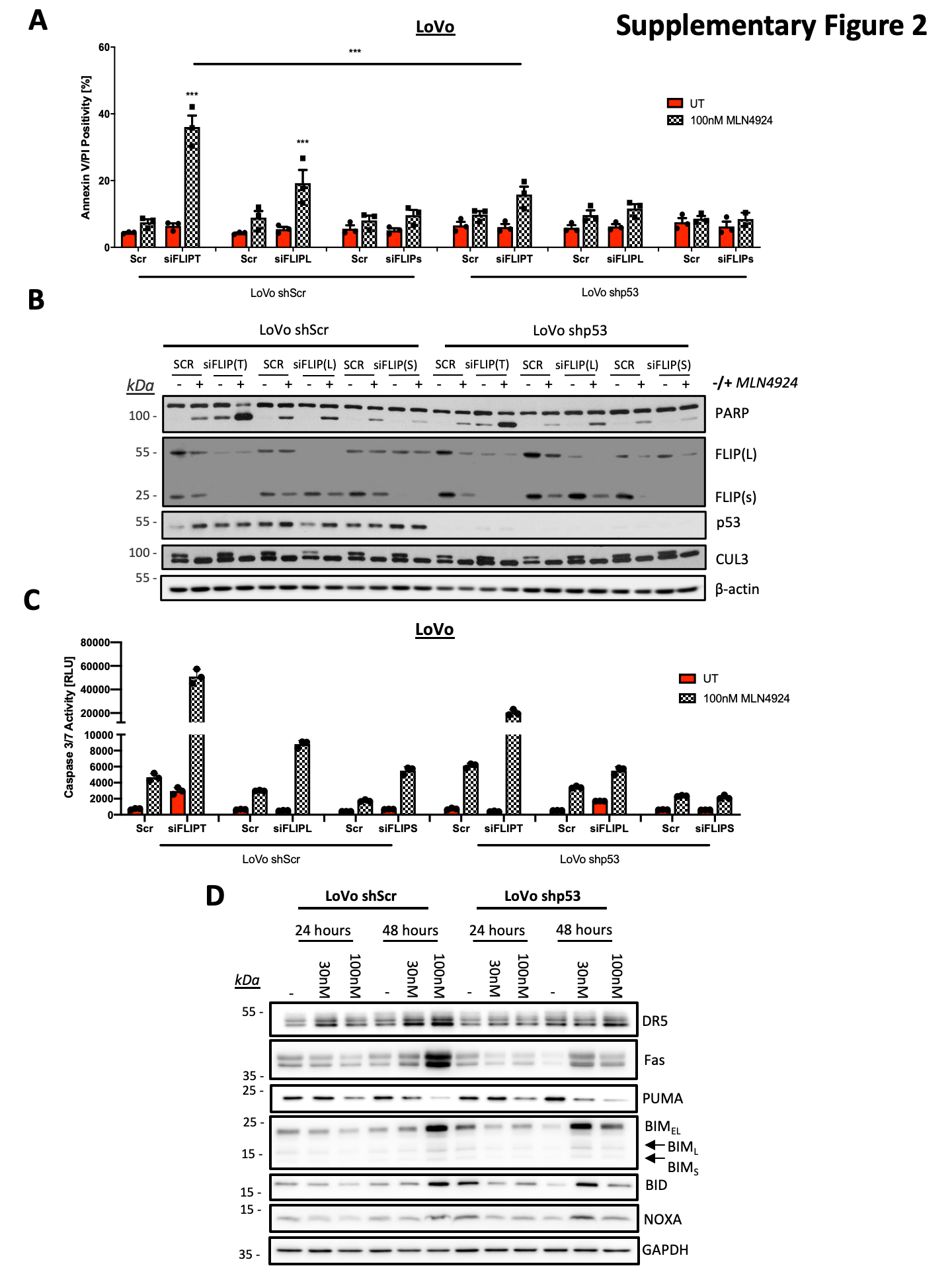

Supplement: Supplementary file 2 — Supplementary Figure 2 [file 41420_2020_296_MOESM2_ESM.png]

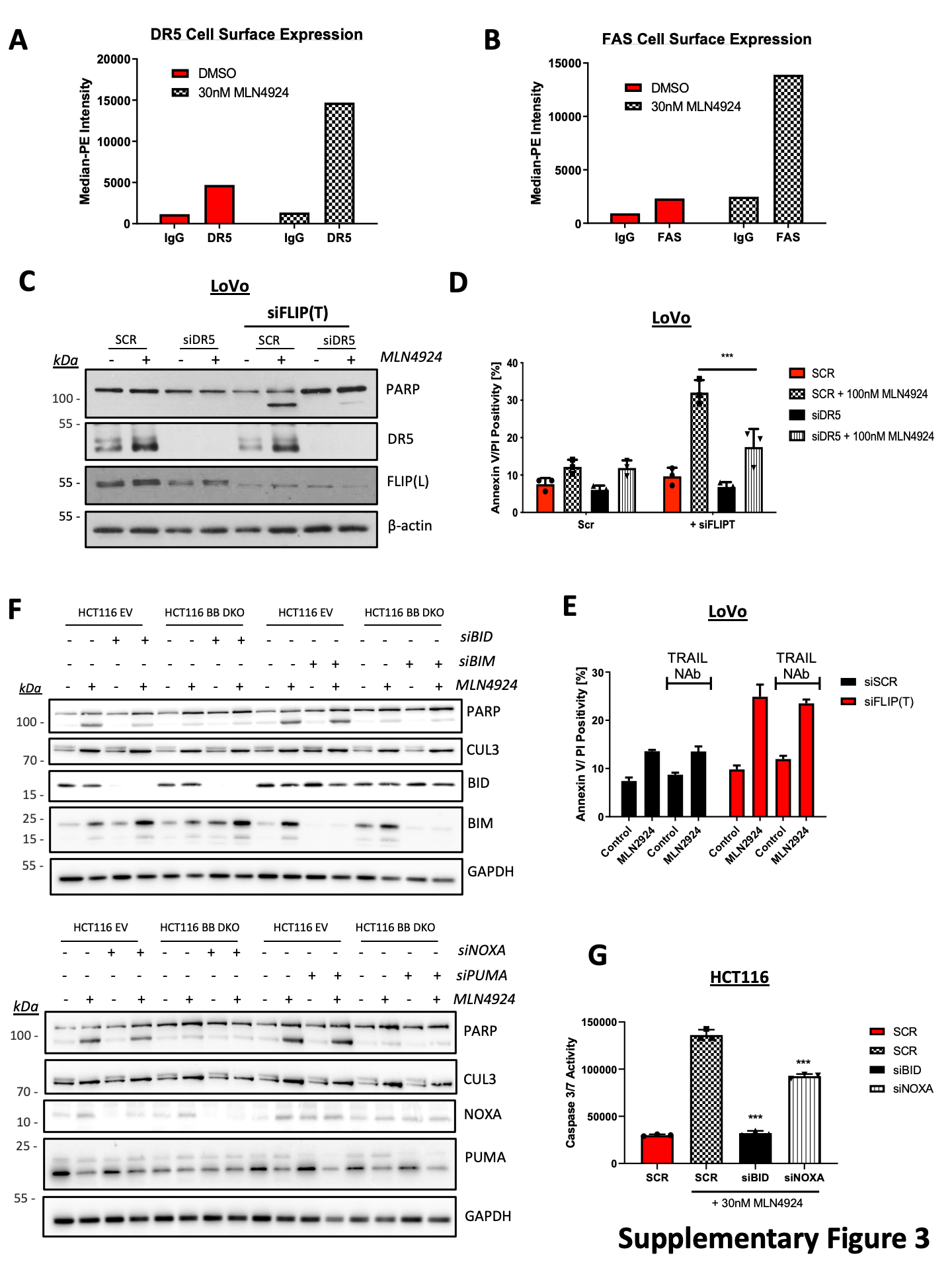

Supplement: Supplementary file 3 — Supplementary Figure 3 [file 41420_2020_296_MOESM3_ESM.png]

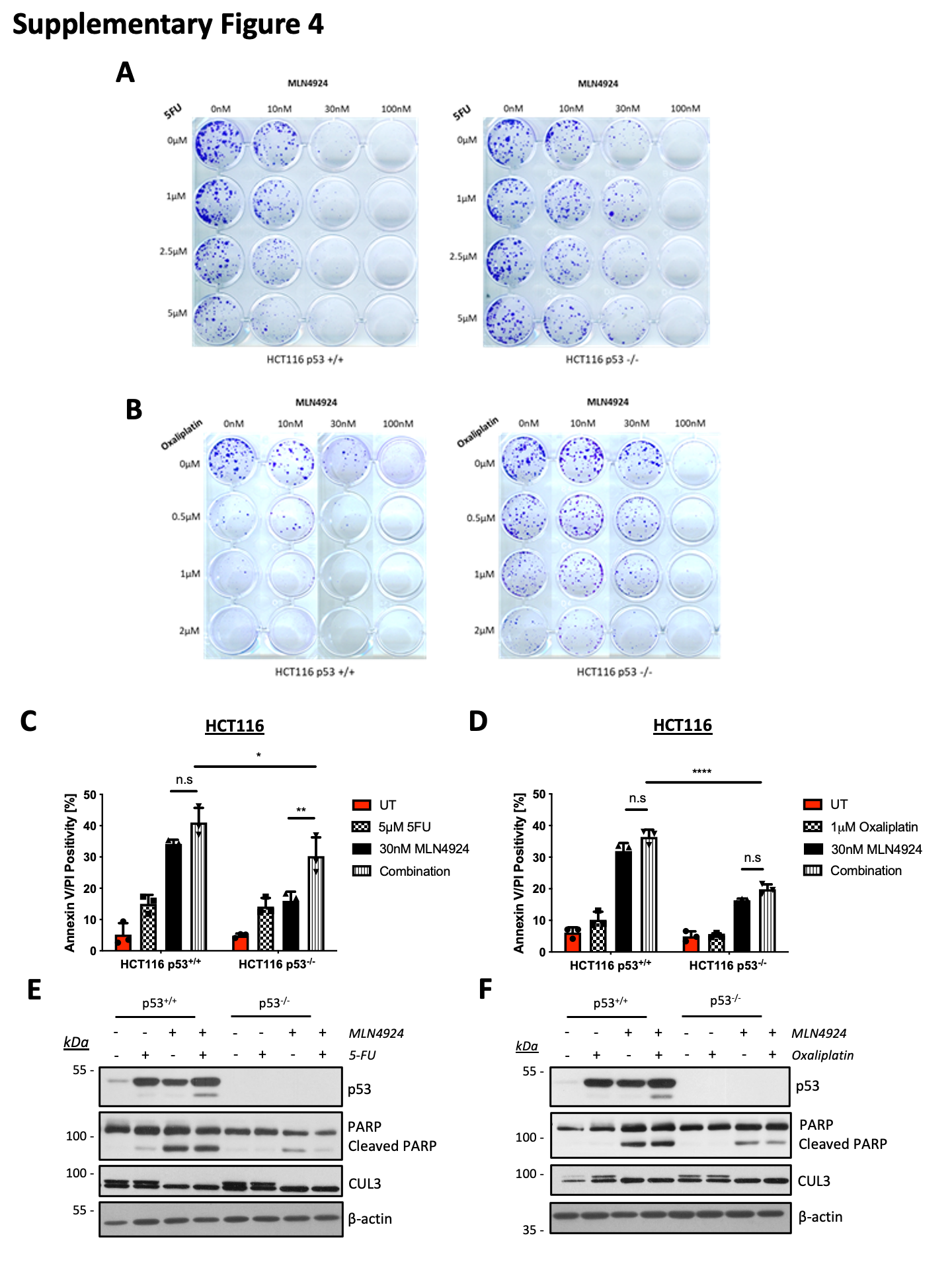

Supplement: Supplementary file 4 — Supplementary Figure 4 [file 41420_2020_296_MOESM4_ESM.png]

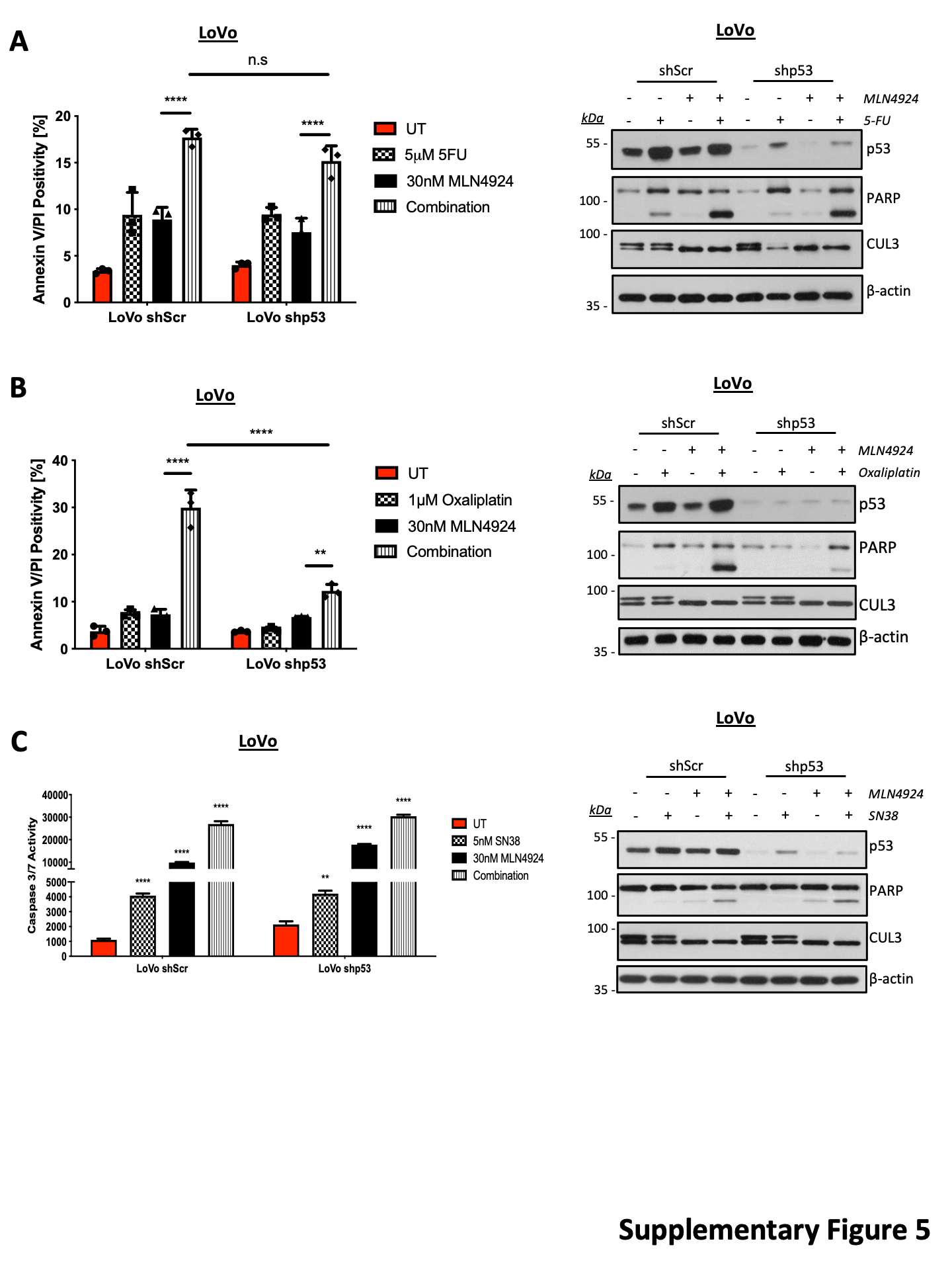

Supplement: Supplementary file 5 — Supplementary Figure 5 [file 41420_2020_296_MOESM5_ESM.png]
