# Supplementary material for: Pevonedistat (MLN4924): mechanism of cell death induction and therapeutic potential in colorectal cancer
Source: Cell Death Discov. 2020 Jul 21;6:61. doi: 10.1038/s41420-020-00296-w (PMC7374701; doi:10.1038/s41420-020-00296-w)
Supplement: Supplementary file 9 — Supplementary Table S4 [file 41420_2020_296_MOESM9_ESM.docx]

**Supplementary Table 4**

| **Antibodies** | **Vendor** | **Catalog Number** |
| --- | --- | --- |
| Anti-Mouse HRP | Cell Signaling Technology | 7076 |
| Anti-Rabbit HRP | Cell Signaling Technology | 7074 |
| BAX | Cell Signaling Technology | 2774 |
| BAK | Cell Signaling Technology | 3814 |
| BID | Cell Signaling Technology | 2002 |
| BIM | Cell Signaling Technology | 2933 |
| β-actin (AC-74) | Sigma | A5316 |
| Cleaved Caspase-3 | Cell Signaling Technology | 9662 |
| Caspase-8 (12F5) | Enzo | ALX-804-242-C100 |
| Cullin3 (3) | BD Biosciences | 611848 |
| DR5 (D4E9) XP | Cell Signaling Technology | 8074 |
| FAS/CD95 | Cell Signaling Technology | 8023 |
| FLIP (NF6) | AdipoGen | AG-20B-0056-C100 |
| GAPDH (6C5) | abcam | ab8245 |
| NOXA | Cell Signaling Technology | 14766 |
| PARP | Cell Signaling Technology | 9542 |
| PUMA | Cell Signaling Technology | 4976 |
| p21 (C-19) | Santa Cruz Biotechnology | sc-397 |
| p53 (DO-1) | Santa Cruz Biotechnology | sc-126 |
| PE Mouse IgG1, κ Isotype Ctrl | Biolegend | 400111 |
| PE anti-human CD262 (DR5, TRAIL-R2) | Biolegend | 307405 |
| PE anti-human CD95 (Fas) | Biolegend | 305607 |
